# Experimental investigation of a viscoelastic liner to reduce under helmet overpressures and shock wave reflections

**DOI:** 10.3389/fbioe.2024.1455324

**Published:** 2024-08-30

**Authors:** Cody J. H. Thomas, Fatih Dogan, Catherine E. Johnson

**Affiliations:** ^1^ Mining and Explosives Engineering Department, Missouri University of Science and Technology, Rolla, MO, United States; ^2^ Materials Science and Engineering Department, Missouri University of Science and Technology, Rolla, MO, United States

**Keywords:** blast injury, combat helmet, shock impedance, free-field blast, overpressure

## Abstract

**Introduction:**

Shock wave overpressure exposures can result in blast-induced traumatic brain injury (bTBI) in warfighters. Although combat helmets provide protection against blunt impacts, the protection against blast waves is limited due to the observed high overpressures occurring underneath the helmet. One route to enhance these helmets is by incorporating viscoelastic materials into the helmet designs, reducing pressures imposed on the head. This study aims to further investigate this mitigation technique against under-helmet overpressures by adding a viscoelastic liner to the inside of a combat helmet.

**Methods:**

The liner’s effectiveness was evaluated by exposing it to free-field blasts of Composition C-4 at overpressures ranging from 27.5 to 165 kPa (4 - 24 psi) and comparing shock waveform parameters to an unlined helmet. Blasts were conducted using an instrumented manikin equipped with and without a helmet and then with a helmet modified to incorporate a viscoelastic liner. Evaluation of blast exposure results focused on the waveform parameters of peak pressure, impulse and positive phase duration.

**Results:**

The results show that peak overpressure was higher when wearing a helmet compared to not wearing a helmet. However, the helmet with the viscoelastic liner reduced the average peak overpressures compared to the helmet alone. For the lowest overpressure tested, 27.5 kPa, the helmet liner decreased the overpressure on the top of the head by 37.6%, with reduction reaching 26% at the highest overpressure exposure of 165 kPa. Additionally, the inclusion of the viscoelastic material extended the shock waveforms’ duration, reducing the rate the shock wave was applied to the head. The results of this study show the role a helmet and helmet design play in the level of blast exposure imposed on a wearer. The testing and evaluation of these materials hold promise for enhancing helmet design to better protect against bTBI.

## 1 Introduction

The combat helmet is a vital piece of military equipment that has increased warfighter survival since its inception by creating a physical barrier between the wearer and kinetic threats. During the twentieth century, the combat helmet progressed from protection against artillery shell fragments (Carey et al., 1982) to defeating direct impacts from 9 mm bullets (Tham et al., 2008). Today, researchers are working to protect the modern warfighter from the threat of traumatic brain injury (TBI). In 2021, a total of 18,773 TBIs were reported by the Department of Defense (DOD) (DOD, 2021). Of particular note is blast-induced TBI (bTBI), which has been described as an occupational hazard as warfighters may be exposed to repeated low-level blasts during training and deployment (Belding et al., 2021a). A sound understanding of shock waves that could cause bTBI can lead to helmet design improvements which protect the warfighter in training and combat, increasing force readiness.

A limitation of modern helmets in preventing bTBI is the underwash effect. In this phenomenon, areas under the helmet experience higher overpressure due to interactions of the shock wave between the head and helmet as the wave moves over the head and gets trapped underneath the helmet (Azar et al., 2020; Hosseini-Farid et al., 2020; Li et al., 2020; Skotak et al., 2020). Blast exposures either high or low can lead to bTBI in some metric depending on the severity and proximity (Belding et al., 2021b), therefore reducing parameters related to blast exposure such as overpressure could help reduce the prevalence of bTBI. As shock wave-helmet-head interactions are becoming better understood, it is essential to develop technologies that reduce under-helmet overpressures and enable helmets to provide better protection.

Time-pressure waveforms illustrate shock wave exposure imposed on a warfighter. Shock waveforms can be evaluated with the following characteristics: peak overpressure, rise time, waveform duration, and impulse (Belding et al., 2021c; WBDG, 2008). To define those characteristics, peak pressure is the maximum pressure of a shock wave. Rise time shows how fast the peak pressure is reached after the pressure rises above ambient and can indicate the rate of loading during the interaction between the shock wave and the object. The duration is the length of time of the first positive phase, determined by the difference in time from the first increase above ambient pressure to the beginning of the negative phase of the waveform. Lastly, impulse is found by taking the integral of the positive phase of the time-pressure waveform. This would be the total amount of force put on the head. Importantly, these characteristics will change depending on the setting and source of the shock wave generated, meaning a breaching charge will have a different waveform compared to firing a mortar. Wearable sensors can help track this information to give information to potentially understand the injury to the warfighter (Wiri et al., 2023). Ideally, reduction of peak pressure, duration, and impulse result in less force applied to the warfighter and buildings around them, thus being a less harmful exposure for the warfighter. It should be noted a less harmful exposure may not truly indicate the precise amount of brain damage that was prevented and injury mechanisms are still an active research area. Nonetheless, solutions that can achieve this should be further studied.

A potential solution which researchers have examined has been to adapt helmets to divert shock wave flow, thus preventing waves from getting under the helmet. Suggested modifications include incorporating thick foam pads inside the helmet, thereby creating a seal (Ganpule et al., 2012), and using a visor and mandibles to shield the front (Mott et al., 2014). Another consideration in helmet designs concerns blast directionality (Mott et al., 2014), as some studies have found helmets can protect from overhead blasts. (Op ‘t Eynde et al., 2020). Applying this is difficult as the direction in which the shock wave will come from cannot always be predicted during combat and training situations, so more solutions should be considered.

Another solution considered is to add dampening materials to the helmet (Barsoum and Barsoum, 2015; Barsoum, 2015). Through numerical simulation, polymeric and aluminum foam liners inside helmets were determined to be good candidate materials for reducing overpressure (Lockhart and Cronin, 2015). Moreso through gas gun tests, viscoelastic materials have been tested for dampening loads from shock waves (Bartyczak and Mock, 2014; Barsoum, 2015). In addition, conclusions from an experimental study recommended that a coating of polyurea on the outside of a helmet along with soft viscoelastic layers in the interior materials could dampen shock wave effects (Bartyczak and Mock, 2015). This idea was attempted in an experimental study which coated the top of a helmet with polyurea and observed a reduction in impulse on the head (Dudt et al., 2015).

Viscoelastic materials dampen vibrations and shock waves by converting mechanical energy into heat, a process characterized by hysteresis and frequency-dependent damping (Lakes, 1998). This results in a reduction in the magnitude of mechanical disturbances. This process is dependent on the viscoelastic materials’ complex modulus, which varies with the frequency it’s subjected to, thus determining the extent of damping provided. For this reason, viscoelastic materials are mechanically tested for their vibration suppression (Wang et al., 2022). This is not a complete synopsis of the applications and tests of viscoelastic materials but shows their promise. Therefore, more practical studies are warranted to assess these promising materials further using real-world experimentation. Thus, this would mean conducting free-field high explosive tests would be most representative of training or combat environments where shock waves can dissipate freely in all directions. This would eliminate potential shock-tube testing side effects, such as vortex rings being created and impacting the sensors (Needham et al., 2015) on the outside of the tube, or extending the exposure duration on the inside.

To further investigate helmet designs to reduce shock wave loading on the head, free-field tests were conducted using piezoelectric pressure sensors in the skull of a surrogate model of a human head and torso to gather overpressure data on the surface of the head. This surrogate model was exposed to Composition C-4 (C-4) free-field blasts ranging from 27.5 kilopascals (kPa) (4 pound per square inch (psi)) to 165 kPa (24 psi) in 27.5 kPa (4 psi) increments. The methodology used 4 psi (27.5 kPa) increments for a US Military audience, but the results and analysis will be presented in metric units. Tests were conducted without a helmet, with a military helmet, and with a modified military helmet to include an interior viscoelastic liner. This test and evaluation study resulted in time-pressure data, analyzed for peak overpressure, duration, rise time, and impulse.

## 2 Materials and methods

This test and evaluation study used free-field blasts to evaluate protection from shock wave loading on the head provided by a helmet as well as a modified helmet with a viscoelastic liner of Ecoflex 00-30, a two part silicone rubber (Smooth-on) (Smooth-On, 2023). This viscoelastic material was chosen from an internal study, and for its ease of purchase. This internal study examined the reduction in reflected shock wave velocity from the following commercially available materials: Ecogel, Ecoflex, Vytaflex, and PMC 121/30 compared to a Kevlar plate. Ecoflex had one of the highest reductions in reflected shock wave velocities and was consequently selected for this study. Other viscoelastic materials could be used in future tests to consider weight, rigidity, and other parameters. This series of air blasts was designed to expose an instrumented manikin to predicted overpressures. The predicted pressures experienced ranged from 27.5 kPa, similar to that created in breaching, to 165 kPa, comparable to an improvised explosive device (IED) event, in increments of 27.5 kPa. This method allowed for the shock wave interactions on different parts of the head to be evaluated using shock parameters of overpressure, rise time, duration, and impulse.

### 2.1 Free-field experimental method

**FIGURE 1:**
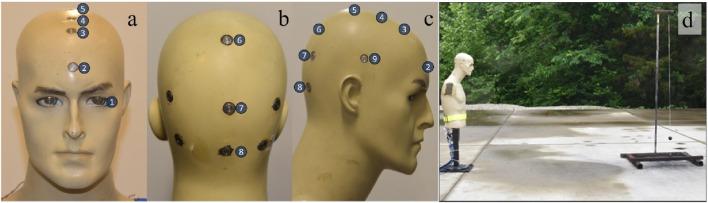
Sensor locations on the manikin (note that there are more sensors present on the manikin than were used in this study): **(A)** sensor 1, the left eye, and sensors 2–5, from the forehead to the top of the head. **(B)** the back of the head, sensor 6–8 locations. **(C)** sensor 9, the side of the head. **(D)** The set up of the manikin bolted into the ground facing the charge.

A surrogate model was constructed from the torso and head of a manikin model MM-BC8S (Mall, 2022) with the following modifications: holes were drilled into the head, metal threads were epoxied on the inside, and PCB Piezotronics Model 102B18 (PCB Piezotronics, 2022) and 102B15 high-frequency Integrated Circuit-Piezoelectric (ICP) sensors were mounted flush with the exterior of the head. The sensor wires were fed out the back of the torso for connection to a Synergy Hi Techniques data acquisition system Synergy P (DAS) (Hi-Techniques - Synergy P, 2022), with a sampling rate of 1 MHz bandwidth. The sensor locations are shown in Figure 1, along with a full image showing the torso in the test set up. The manikin was filled with Clear Ballistics ballistic gel (Clear ballistics, 2022) to give the interior a flesh-like density. The torso was mounted onto a metal stand capable of bolting to the ground. The manikin’s head height was 23 cm, its breadth was 14 cm, head circumference was 53 cm.

**FIGURE 2:**
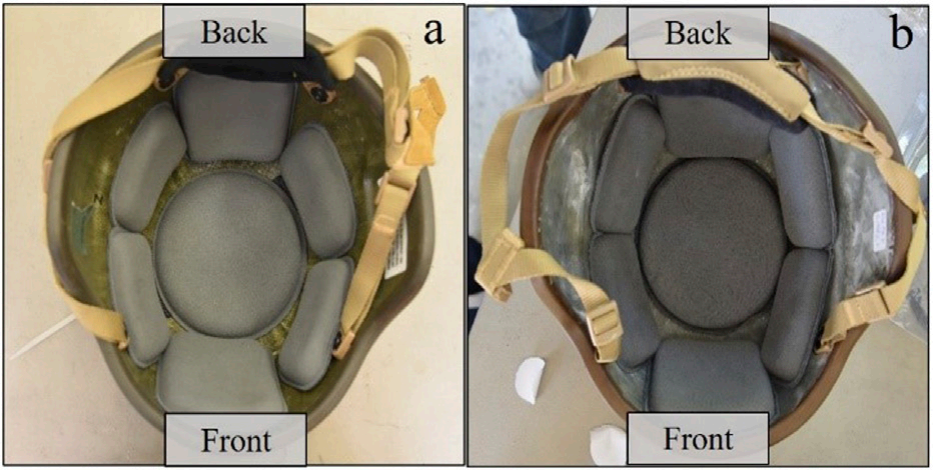
Helmet pad orientation on the inside of the helmet. **(A)** helmet, and **(B)** modified helmet with liner.

The helmet straps were securely tightened onto the manikin’s chin. The helmet pad orientation for both helmet regimes is shown in Figure 2, based on the helmet operator’s technical manual (TM 10-8470-204-10, 2008). Care was taken to ensure the helmet pads were touching the manikin head. The clear viscoelastic material added 200 g (g) to the helmet, resulting in a total weight of 1446 g, a 16% weight increase. While casting the liner into the helmet, variations in thickness occurred. The majority of the helmet liner had a thickness ranging from 1 to 2.5 mm, while some small sections reached a thickness of 6 mm. A 3D scan of the liner is provided in Figure 1 of the Supplementary Material.

During a suspended air blast, ground reflected waves complicate waveform profiles. Interaction between the incident and ground reflected waves creates a triple point with the Mach stem below. Lowering the charge height relative to the target increases the height of the triple point, enabling just the Mach stem to interact with the target, thus simplifying the resultant waveforms. To make this simplification, the charge height and mass were adjusted for the desired side on pressure. For detailed methodology, see our previous work (Swisdak, 1975) following data from Swisdak (Thomas and Johnson, 2024). Data from these sources also allowed the distance and charge size to be chosen to predict overpressures at the top mid-line of the head (sensor 4), presented in Table 1. The range of overpressures was chosen to encompass the safety standard threshold for the U. S Army of 27.5 kPa (4 psi) and an IED event of 165 kPa (24 psi) in increments of 27.5 kPa (4 psi). Pressures for the forthcoming analysis are presented in kPa rather than psi. Table 1 lists the predicted side-on pressures, Composition C-4 (C-4) charge size, Trinitrotoluene (TNT) equivalence based on overpressure (Jeremi and Baji, 2006), distances from the manikin, and charge height. Three tests were conducted at each overpressure: without the helmet, with a helmet, and with a modified helmet with viscoelastic liner.

**TABLE 1:** Charge sizes, predicted pressures, distance of the charge from the manikin, and height of the charge.

Predicted side on pressure (kPa/psi)	Charge size C-4 (g)	TNT equivalence (g)	Distance from manikin (m)	Height of charge (m)
27.5/4	41	55	2.5	0.3
55.1/8	140	189	2.5	0.45
82.7/12	135	182	2	0.3
110/16	204	276	2	0.3
138/20	281	380	2	0.3
165/24	360	486	2	0.3

The sensors unprotected by the helmet and facing the blast head on were expected to receive a total reflected overpressure, whereas those on top and side of the head would receive a side on overpressure. The sensors protected by the helmet would receive a combination of head on and side on overpressures as well as oblique angle reflected waves caused by helmet-head reflections. In addition to the aforementioned overpressure data collection, a monochrome Phantom V2012 (Phantom V2012, 2022) high-speed camera recording at 1,00,000 frames per second documented the blast. Overpressure data was analyzed for peak pressure, duration, rise time, and impulse. Statistical tests were not performed due to the limited number of trials, but the root mean square formula was used for overall trends. Normalization of the data was performed to remove any environmental variances. Phantom camera control (PCC) software was calibrated and used to measure the movements of the helmet in the footage, and shock waveforms were visually examined.

## 3 Results and discussion

### 3.1 Normalization

**FIGURE 3:**
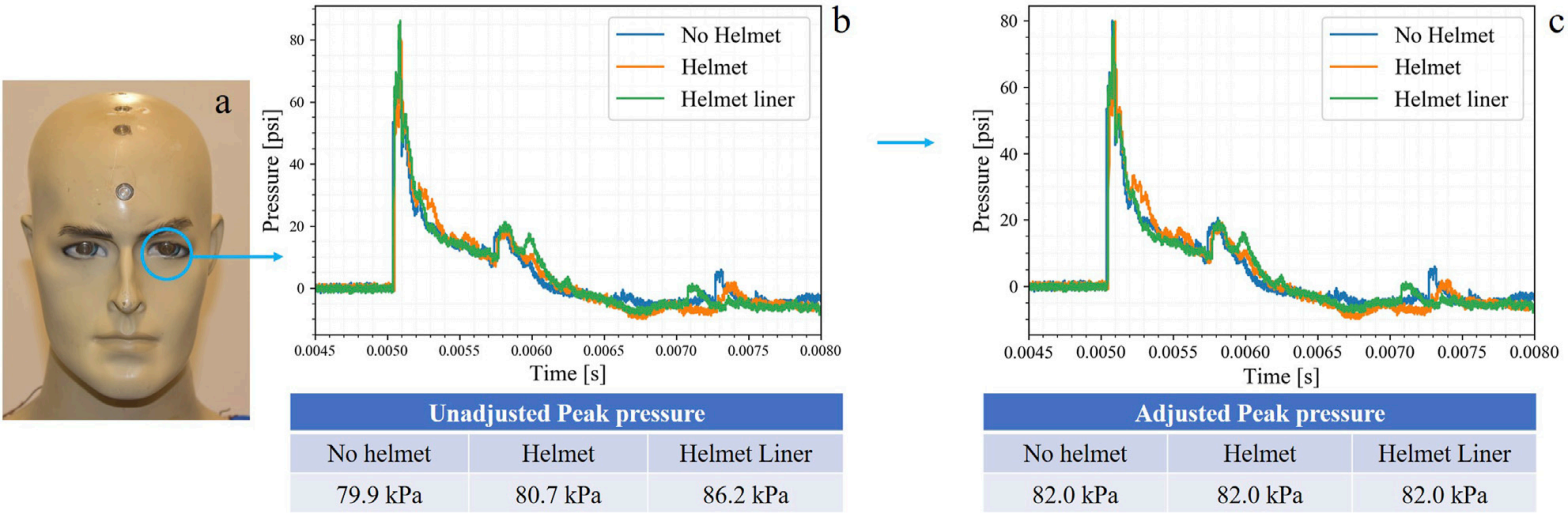
27.5 kPa (4 psi) blast exposure waveforms. **(A)** sensor 1 location used to adjust data, **(B)** original waveforms with unadjusted data **(C)** adjusted waveform with adjusted data.

Eighteen free-field blasts were conducted and analyzed using time-pressure waveform data to evaluate shock wave behavior at different parts of the head using no helmet, helmet, and modified helmet liner regimes. Slight variations in pressure data were observed during the test period, attributed to changing environmental conditions such as humidity, wind, and air temperature, as well as charge configuration factors like density, geometry, and mass which are typical in free-field blasts. To account for these effects, an average peak pressure from sensor 1, the eye, in all three scenarios, was taken and used as a multiplier correction factor to adjust values for all waveforms in each overpressure exposure. An example of this adjustment is shown in Figure 3.

### 3.2 Shock loading on the head

**FIGURE 4:**
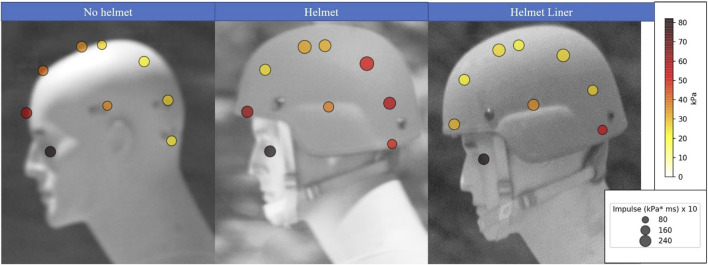
Heat Map of the 27.5 kPa (4 psi) blast exposure showing the peak pressure based on color and impulse based on the size of the head at sensors without a helmet, with the helmet, and with the helmet and the viscoelastic helmet liner.

**FIGURE 5:**
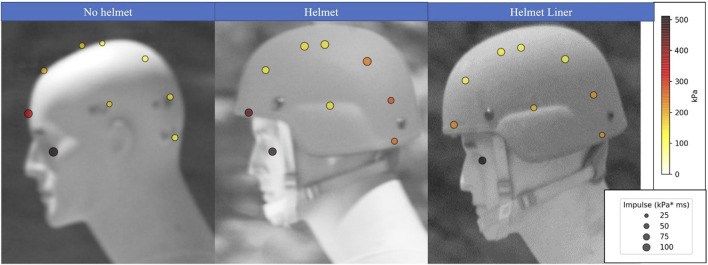
Heat Map of the 165 kPa (24 psi) blast exposure showing the peak pressure based on color and impulse based on the size of the head at sensors without a helmet, with the helmet, and with the helmet and the viscoelastic helmet liner.

Exposure heat maps were created in Python (Matplotlib, 2023) to present a comparison of peak overpressures and impulses from separate sensor locations (sensor locations are shown in Figure 1) to evaluate shock loading on different parts of the head. With increased overpressures, and impulses being the metric to cause more injury to the head. These aspects for a 27.5 kPa (4 psi) and 165 kPa (24 psi) blast exposure with no helmet, helmet, and helmet liner are shown in Figures 4, 5, respectively. The pressure at sensors 1–9 can be compared using a color grade scale, while the impulse is visually compared using a circle area scale. Scales are shown to the right of Figures 4, 5 with different magnitudes for each heat map. The peak pressures and impulses used to create these heat maps can be found in the tables in the Supplementary Material.

For the 27.5 kPa exposure (Figure 4), the peak overpressure at the top of the head (sensor 5) with a helmet liner was 21.2 kPa, a 21% decrease in compared to no helmet (26.2 kPa), and a 37.6% decrease compared to using the helmet (31 kPa). For the highest tested exposure of 165 kPa (Figure 5), peak overpressure at the top of the head with a helmet liner was 113.3 kPa, a 4.5% decrease in compared to no helmet (118.6 kPa), and a 26% decrease compared to using a helmet (147.2 kPa). Predictably, the eye received the highest peak pressure, as it was unprotected and directly facing the blast, thus received a head-on pressure when the mannikin was in this orientation. If the mannikin was turned 90° away from the blast, the eye would have received a lower side-on pressure. Additionally, the shape of the face around the eye may act to funnel the shock wave into the sensor. This highlights the importance of wearing eye protection not only against fragmentation and particulates, but also during blast exposure.

Peak pressure and impulse were notably higher on the back of the head when a helmet or helmet liner were present compared to tests without them, likely due to shock waves under the helmet colliding after they have moved over and around the head. An additional collision also occurs as the shock wave reflects off the shoulder upwards and into the helmet. These collisions are known to cause a higher overpressure upon interaction with each other than the sum of the separate overpressures (Cooper, 1996). Examining the shock wave overpressures with the helmet during the 165 kPa exposure show an overpressure of 155 kPa on the side of the head (sensor 9), whereas overpressure reached 269 kPa at the back of the head (sensor 7) due to shock waves colliding. Conversely, when no helmet was worn, the back of the head experienced a much lower overpressure of 147 kPa. When incorporating the helmet liner, peak pressure reached 205 kPa at the back of the head, a decrease of 27% relative to the helmet. Interestingly, the side of the head (sensor 9) experienced a slightly higher overpressure with the helmet liner of 173 kPa compared to the helmet (155 kPa). This deviation from the trend may be due to some data scatter at this high-pressure exposure, but without statistical analysis was not removed as an outlier.

The increased overpressure at the back and top of the head with helmets on compared to no helmet being worn described in the heat maps indicate that shock wave collisions occurred. This outcome is in line with data from another study which observed the under-wash effect from a variety of modern combat helmets, and compared overpressure data to a no helmet scenario (Skotak et al., 2020). However, the helmet liner overpressure decrease indicates that dampening from the viscoelastic material did occur. Initial inspection may cause the reader to consider dampening to occur locally at different parts of the head, however, because viscoelastic materials are made up of polymer chains, force interaction at one part of the polymer will affect the rest of the linked chains, leading to a possibility that the entire mass of the viscoelastic liner was causing this dampening effect.

As no replicate data was collected, general commentary on the differences between test regimes rather than statistical differences were described with use of the heat maps in Figures 4, 5. However, to provide more insight into the data collected on these overpressure exposures, the root mean squared (RMS) method, shown in Equation 1, was employed for orthogonally orientated sensors. This would allow a more thorough search for trends in the front (sensor 2), top (sensor 5), and side (sensor 9) compared to the back (sensor 7), top and side sensor waveform data. Higher overpressure, and impulse would indicate a higher likelihood of injury to the head, with the effects of duration described later on in the analysis.

$$RMS=\sqrt{\frac{1}{n}\sum_{i}x_{i}^{2}}$$ (1)

Calculations were performed where “n” represented the number of measurements (three in our case, corresponding to the front, top, and side of the head, or the back, top, and side of the head), and “x” denoted the waveform parameter value from head’s respective area. Results are presented in Tables 2–4 for peak pressure, impulse, and positive phase duration, respectively. Rise time was not evaluated using this method but was included in analysis later when examining the shock waveforms. These RMS evaluations for no helmet, helmet, and helmet liner focused on the peak pressures and impulse, using the durations to provide context for the impulse values. Subsequently, heat maps were generated using these values and are presented in Tables 2–4, the lowest (green), intermediate (yellow), and high (red) values for each designed pressure level are highlighted. Front, top, and side RMS values are labeled as “front”, and the back, top and side values are labeled as “back”. This combined analysis offers insight into the force directed to the head from the shock wave in the tested scenarios.

**TABLE 2:** The RMS equation was applied to the peak pressure values collected for different test regimes and parts of the head.

	Front			Back		
Designed pressure level (kPa)	No helmet	Helmet	Helmet liner	No helmet	Helmet	Helmet liner
27.5	42.1*	44.8*	29.7*	28.9	42.7	28.3
55.1	77.2*	85.5	51.7*	52.4	87.6*	44.8
82.7	87.6*	124.8*	84.1*	68.3	101.9	71
110	150.2*	157.0*	182.1*	126.9	126.8	99.3
138	193.1*	218.3*	178.6*	129.6	166.2	147.6
165	232.1*	278.9*	179.0*	146.9	198.4	170.3

Legend (kPa): 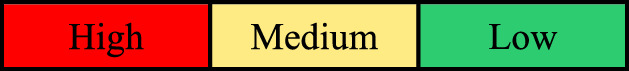

*Higher value when comparing front and back.

A legend indicates differences in the magnitude of overpressure within each designated pressure level. Values of the higher magnitude comparing the front and back of the head are indicated with an * to show the difference, if values were equal then neither have an *.

**TABLE 3:** RMS equation applied to the impulse values collected for different test regimes and parts of the head.

	Front			Back		
Designed pressure level (kPa)	No helmet	Helmet	Helmet liner	No helmet	Helmet	Helmet liner
27.5	17.9*	24.1	20.7	17.2	24.8*	21.4*
55.1	31.0*	57.2	39.3	30.3	60.1*	42.7*
82.7	49.7*	55.2	49.7	48.9	57.9*	49.7
110	61.4*	65.5	60.7	58.6	68.9*	64.8*
138	62.7*	80.7	82.7*	54.5	85.5*	82.1
165	81.4*	99.9	86.9*	60.7	106.2*	82.7

Legend (kPa): 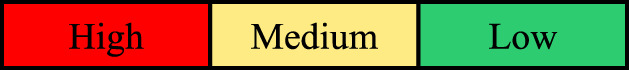

*Higher value when comparing front and back.

A legend indicates differences in magnitude of impulse exposure within each designated pressure level. Values of the higher magnitude comparing the front and back of the head are indicated with an * to show the difference, if values were equal neither have an *.

**TABLE 4:** RMS equation applied to the duration values collected for different test regimes and parts of the head.

	Front			Back		
Designed pressure level (kPa)	No helmet	Helmet	Helmet liner	No helmet	Helmet	Helmet liner
27.5	4.3	4.1	4.3	4.7*	4.5*	4.6*
55.1	5.0	3.7	4.4	5.6*	4.2*	4.6*
82.7	2.8	3.3	3.3	3.6*	3.8*	4.2*
110	3.1	3.0	3.1	3.7*	3.7*	4.0*
138	2.7	3.1	3.4	3.2*	3.6*	3.9*
165	3.6	2.0	2.0	4.2*	2.7*	2.0

Legend (kPa): 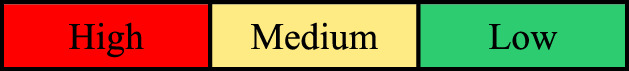

*Higher value when comparing front and back.

A legend indicates differences in the magnitude of the duration within each designated pressure level. Values of the higher magnitude comparing the front and back of the head are indicated with an * to show the difference, if values were equal then neither have an *.

Observations from Table 2 show clear trends in peak overpressure, with nearly all the greatest peak overpressures occurring using the helmet regime, indicated by the red cells. Trends indicate the viscoelastic liner reduced peak overpressure compared the helmet and no helmet regimes. In the helmet liner regime, eight occurrences were noted where peak overpressures were lower than both the helmet and no helmet regimes. These trends were not seen in the 110 kPa blast exposure, potentially attributed to testing variance. For this reason, pressure regime 110 kPa was chosen to be examined further later in the analysis.

Overall, the underwash effect was observed here as pressure increased due to the presence of a helmet, notably in the back of the head. When a comparison of the front versus the back of the head was conducted, it showed that the front had higher overpressures than the back. This trend was evident for all the regimes, except in one instance of the 55.1 kPa exposure, by a narrow margin. This difference in overpressure was likely a result of the reduced distance between the front of the head to the blast compared to the back. The front sensors, when a helmet was not worn, received a head-on impact of the shock wave, while the back sensors received an oblique reflection impact as the shock wave engulfed the head. When a helmet, and helmet liner were worn this trend persisted, but front sensors did not receive a head on impact but a combination of multiple oblique reflection impacts, making proximity a likely cause of overpressure differences. Lastly, to understand the damage these overpressures caused during these exposures the impulse was examined in Table 3.

Analysis of Table 3 shows the lowest impulses occurred without a helmet worn except in two cases. These two cases were the 82.7 and 110 kPa exposures, where in the 82.7 exposure, the no helmet and helmet liner both had the lowest impulses comparing by the tenth decimal place. The highest impulses occurred throughout the helmet regime except in one case. Generally, this follows the trend of the helmet having higher overpressures under the helmet compared to without a helmet. Comparing the two helmet regimes, incorporating the viscoelastic liner reduced the impulse experienced on the head. It should be noted that though overall trends were observed, pressure exposures of 82.7, 110, and 138 kPa did not entirely follow them.

An examination of the impulse in the front versus the back of the head showed the impulse was lower in the back for the no helmet regime, but impulse in the front was lower in the helmet regime. However, the helmet liner had a mixture of these results comparing front to back, thus not indicating a definitive trend. The low impulse in the back of the head for the no helmet regime indicates that the shock wave was able to reflect off the head and the overpressure was not sustained. However, when the helmet was added the shock wave reflecting in the back of the head likely had difficulty escaping from under the helmet leading to a larger impulse. If the case was that the shock wave had a hard time escaping from under the helmet, then this would be shown by examining the positive phase duration of the shock waves as depicted in Table 4.

Based on the duration data, only a few discernable trends were observed. The most notable was that the helmet liner increased the duration of the shock waveforms compared to the helmet. For example, in the 55.1 kPa exposure, the helmet liner’s RMS values of 4.4 and 4.6 were higher compared to the helmet’s 3.7 and 4.2. For the helmet at 27.5 and 55.1 kPa pressure exposures, the lowest durations were observed compared no helmet and helmet liner. The helmet liner’s higher duration may explain why its impulse values were not the lowest despite having some of the lowest overpressure values observed, such as in the 27.5 exposure, with 29.7 and 28.3 RMS values. The highest durations were found to be without the helmet, but in the 27.5 and 110 kPa exposures the duration at the front section without a helmet and with the helmet liner were the highest. To understand why these differences in duration occurred, shock waveforms of the blasts are examined later in the analysis.

The front of the head experienced a lower duration than the back based on these RMS values. This does not completely support the notion that higher duration values show that the shock wave stuck under the helmet as this trend was observed in the no helmet regime. However, comparing the duration values here to the peak pressure values in Table 2 would explain shock wave behavior. Higher overpressure values in the front of the head would indicate higher shock velocities in the front, therefore resulting in shorter durations there, as seen in Table 2. Then the longer durations and lower overpressures at the back of the head show the shock wave slowed down. With the helmet regime, the overpressures were the highest, thus the lowest durations. Considering all RMS results, the shock wave behavior in the helmet liner regime could be explained by the following: a) the shock wave’s travel slowing with diminishing pressure and increasing duration, b) the shock wave being unable to escape from under the helmet, and c) the shock wave unable to move quickly out under the helmet. These behaviors resulted in a generally higher impulse than the no helmet regime.

The duration, unlike overpressure and impulse, does not have explicit expectations on what a lower or higher value means to indicate reduced damage to a subject. A longer duration would indicate more time during blast loading, and possibly more time for the material to manage the load being impressed. However, if this longer duration is coupled with a slower rise time and decay, it could be better than a short duration fast rise time exposure because the rate of injury would be lower. Thus, an argument could be made that longer durations of the no helmet and helmet liner could have caused less damage. A study that examined the rise times and injury in rats (Neuberger et al., 2014) saw an immediate difference in injury between faster and slower rise times, but injury over the long term from fast and slow rise times was found to not be different. As a metric, duration has been shown to be useful for understanding the two common indicative metrics of injury, impulse and peak pressure, but it is rather inconclusive as a metric from a blast injury standpoint. These shock waveform parameters can be further understood by examining the shock waveforms for each exposure.

The shock waveforms from the 27.5, 110, and 138 kPa blast exposures were examined to analyze the general trends described in Tables 2, 3. Other waveforms that are not used in this comparison can be found in the Supplementary Material. The waveforms collected exhibited a negative phase before returning to ambient pressure, but that will not be the focus of this analysis. Instead, only the first positive wave was evaluated. An example of how these graphs were cropped is shown in Figure 3 of the Supplementary Material, presenting the 27.5 kPa waveform.

**FIGURE 6:**
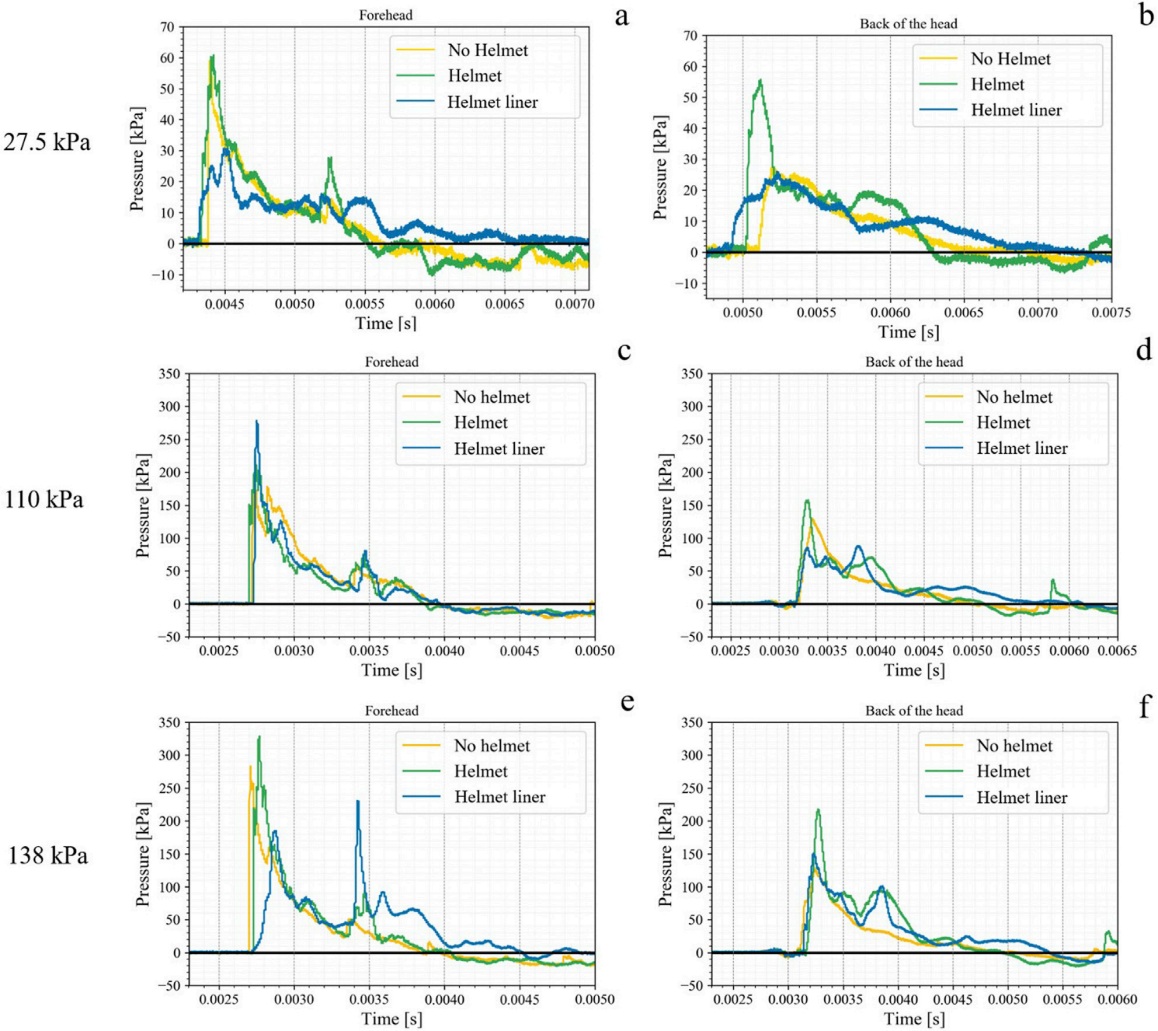
Shock waveforms for the forehead and back of the head for 27.5 **(A, B)**, 110 **(C, D)**, and 138 kPa **(E, F)** blast waveforms.

Examining Figure 6, the waveforms describing the shock wave impact on the forehead reveal that the helmet liner reduced the peak pressure and slowed the rise times compared to the other helmet regime. The majority of rise times were fast; however, in examples such as Figure 6D, the peak pressure for the waveform was after the initial peak pressure, causing the rise time to be longer. The waveforms at the forehead are slightly shorter than those in the back of the head, which indicates slightly slower loading based on the location on the head. This would slow the injury rate on the head. From the data in this study the difference in rise time between these are minimal, making precise comments on the change in rise time due to location of the head challenging. Another notable observation was the duration of the helmet liner versus the helmet regime, which shows an increase in all cases except for Figure 6C, the forehead at 110 kPa. Additionally, in Figure 6C, the helmet liner had a greater peak overpressure than the helmet, which would explain the helmet liner having the highest RMS value at 110 kPa in Table 2. However, the reason the 110 kPa blast exposure had a different pattern was unclear from these waveforms. For the 138 kPa exposure in Figure 6E, a secondary reflection in the helmet liner waveform skewed the data to increase the waveform duration. Due to this, the data in Table 4 showed the helmet liner had the highest positive phase duration for the 138 kPa exposure but the waveform did show dampening during the first peak. The no helmet regime’s waveforms showed a smooth overpressure decay in all overpressure exposures.

### 3.3 Dampening mechanisms

From Tables 2–4, the main output was that it is possible to reduce the loading on the head by adding a viscoelastic liner. The waveforms in Figure 6 show the helmet and helmet liner distort shock waveforms, thus altering the overpressure exposure on the head. This overpressure reduction shown in the helmet liner data could be due to four possible mechanisms: 1) the dampening properties of the viscoelastic material, 2) the reduced air gap between the helmet and the head reducing the possibility for shock waves to enter and travel, 3) the added weight changing how the helmet moves on the head as the shock wave passes, or 4) the added material changes how the helmet sits on the head causing it to tilt slightly forward. This tilt of the helmet is presented for all blast exposures in Figure 2 of the Supplementary Material. This tilt of the helmet with the liner, may have shielded the front of the head slightly more than the helmet without the liner and be the cause of the reduced overpressure experienced on the head. Further investigation into this mechanism in recommended as any small change to reduce overpressure exposure to the wearer is beneficial. The first three mechanisms are expanded on in the following paragraphs.

**FIGURE 7:**
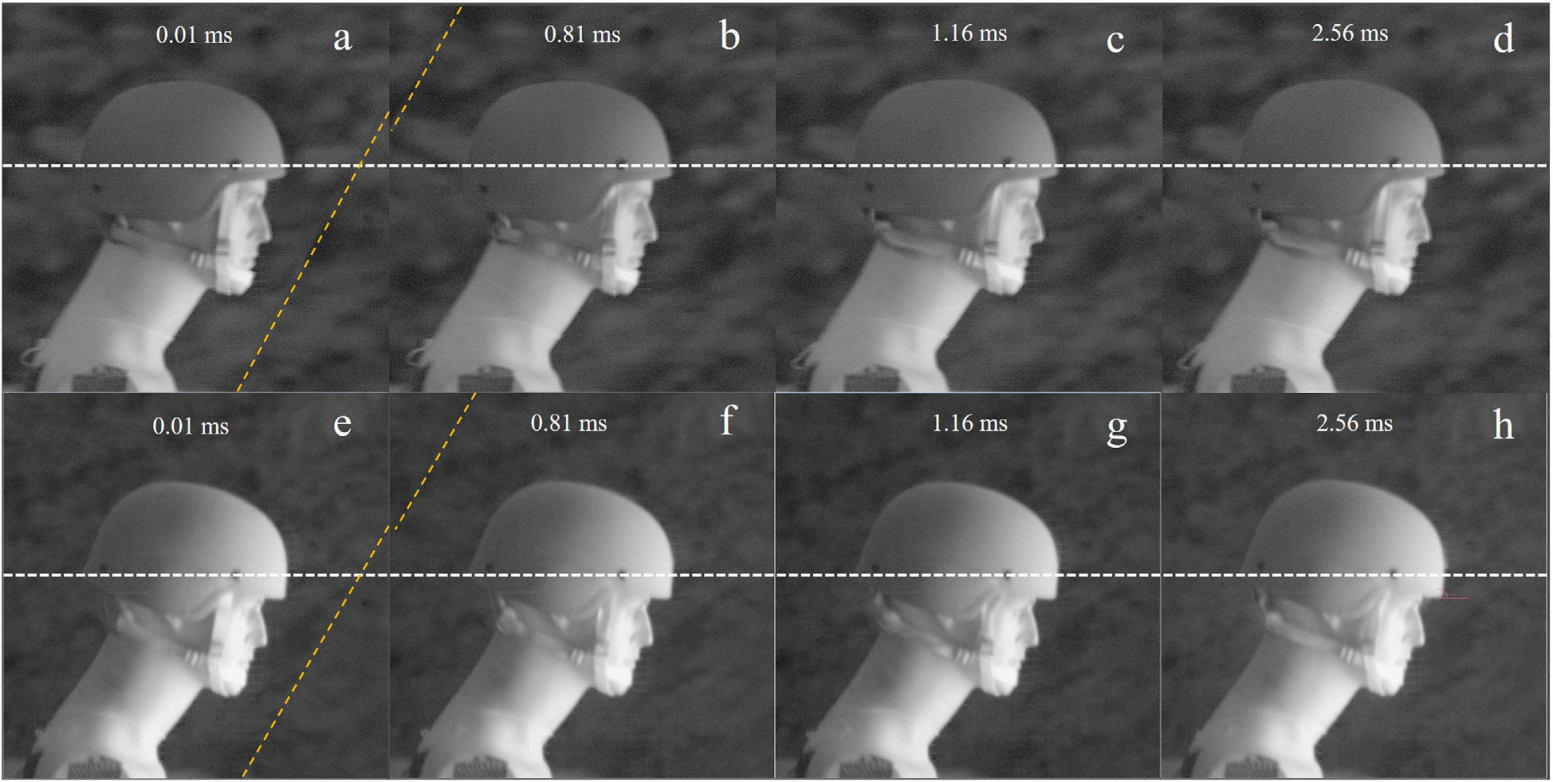
**(A–D)** shock wave progression and momentum transfer for the helmet, and **(E, F)** for the helmet liner regime. **(A, E)** shock wave frame before the impact of the shock wave on the helmet, **(B, F)** is 80 frames where the shock wave has passed the helmet. **(C, G)** Movement of the helmet detected. **(D, H)** frames later, near the end of the footage. A white checkered line is the location of the helmet screw.

High-speed footage was used to examine if the helmet shifted during a blast event, causing a change in overpressure to the sensors. To detect this shifting, the distance the helmet screw moved was measured with the use of PCC software. Comparing the difference in movement of the screw between the helmet and the helmet liner regime helped determine if the center of gravity changed due to increased weight from the liner, possibly affecting overpressure exposure as the shock wave interacts with the head. This was done with the 165 kPa blast exposure footage because, as the maximum amount of force imposed, it would show the most helmet shifting. Frames from the PCC software with the helmet are shown in Figures 7A–D and helmet liner in Figures 7E–H. Wave progression before shock wave passage is shown in Figures 7A, E, and after interacting with the helmet is in Figures 7B, F. The helmet screw was at the same level before and after the frames with the helmet-wave interaction, as seen in the left-hand corner in Figures 7B, F. Movement was detected 35 frames later seen in Figures 7C, G. The measurement of helmet movement started when the helmet started to move, and measuring ended after 140 frames.

This can be seen as the screw of the helmet moved above the dashed line from frame 116 to frame 256. Over 140 frames, the modified helmet moved 2.5 mm, and the helmet moved 2.7 mm. The ruler used to calibrate the PCC active measurement had 0.5 mm marks, thus this small difference could be due to that uncertainty in the measurement. This small difference in shifting could also be due to the increased weight of the helmet liner and may have increased duration very slightly. However, as shown in Figures 7B, F, the shock wave passed the manikin before the helmet started to shift. Therefore, the impact on peak overpressure due to the change in center of gravity would be minimal. For helmet designers, the change in the center of gravity from the helmet liner may have more long-term effects on the warfighter’s neck and body than the peak overpressure exerted on the head.

The other mechanism that may be affecting the results would be the mechanical properties of the viscoelastic material. This study did not characterize Ecoflex, but other studies have characterized the Ecoflex family of silicone materials (Liao et al., 2020; Liao et al., 2021). Based on their findings, researchers have found that Ecoflex 00-30 silicone rubber exhibits significant stress recovery but has long recovery times (Liao et al., 2020), which could potentially make the helmet more comfortable for the wearer under non-blast conditions. While the precise mechanism triggering this reduction in shock wave overpressure and increase in duration cannot be deduced from the present data, the data does indicate that this phenomenon was occurring. However, when subjected to shock loading, this material may demonstrate stress recovery behavior. Overall, the overpressure dampening from Ecoflex could be attributed to viscoelastic dampening and softening dissipation (Liao et al., 2021). From the data found in this study, researchers may explore incorporating a variety of soft viscoelastic materials into future helmet designs studies.

This test and evaluation study confirms, from a practical standpoint, that simple changes can be made to a helmet to reduce the overall shock loading exposed to the human skull. The exact energy dissipation capabilities of the liner that caused the reduction, such as weight, air gap, and material properties, can be further examined. However, the data collected aligns with previous data that proposed tested viscoelastic materials to dampen shock waves (Bartyczak and Mock, 2014; Barsoum, 2015; Bartyczak and Mock, 2015; Dudt et al., 2015). Future research should focus on the uniform application of liner material for consistent results. It also notes the limitation of conducting single trials per pressure exposure, which may introduce outliers or errors not accounted for by environmental adjustments. Furthermore, the added weight of viscoelastic materials is a concern for wearer comfort and safety, indicating that future designs should balance shock wave dampening, ballistic protection, and lightness to avoid straining warfighters’ necks. Future research could examine layering viscoelastic materials to find the optimal combination of layers (Belding et al., 2021b), material, and additives (Lakes, 1998).

## 4 Conclusion

Free-field blast overpressures from 27.5 (4 psi) to 165 kPa (24 psi) in 27.5 kPa (4 psi) increments were conducted to compare not wearing a helmet, wearing a helmet, and wearing a helmet modified with a viscoelastic helmet liner. Peak pressure and impulse were higher with the addition of a helmet but lower with the addition of a helmet liner for all peak pressures and five of the six pressure regime impulses. A notable example being the helmet liner decreased the overpressure on top of the head (sensor 5) by 37.6% in the highest exposure of 165 kPa, and by 26% in the lowest exposure of 27.5 kPa. The results of this test and evaluation study indicate that wearing a helmet indeed increases the overpressure experienced on the head during blasts, while the incorporation of a viscoelastic liner within the helmet mitigates this effect to some extent. The data suggests that although the liner slightly prolongs the duration of the positive waveform on the head, it also contributes to reducing the overall overpressure experienced. This elongation of the waveform may be attributed to the shock wave being partially trapped under the helmet while the helmet itself remains relatively stable, minimizing drastic movement. At lower pressure exposures, the liner kept impulse on the head at or slightly lower than that felt when not wearing a helmet. The findings underscore the potential of viscoelastic materials in enhancing helmet design to better protect against blast-related traumatic brain injuries (bTBI), offering a promising avenue for future research and development in this critical area of protection.

## Data Availability

The original contributions presented in the study are included in the article/Supplementary Material, further inquiries can be directed to the corresponding author.
