# Supplementary material for: Experimental investigation of a viscoelastic liner to reduce under helmet overpressures and shock wave reflections
Source: Front Bioeng Biotechnol. 2024 Aug 30;12:1455324. doi: 10.3389/fbioe.2024.1455324 (PMC11392881; doi:10.3389/fbioe.2024.1455324)
Supplement: Supplementary file 1 [file DataSheet1.docx]

*Experimental Investigation of a Viscoelastic Liner to Reduce Under Helmet Overpressures and Shock Wave Reflections*, **Frontiers in Bioengineering and Biotechnology, section Biomechanics**, Cody Thomas^1^, Fatih Dogan^2^, Catherine E. Johnson^1^; 1) Mining and Explosives Engineering Department, Missouri University of Science and Technology, Rolla, Missouri, 65409, USA 2) Materials Science and Engineering Department, Missouri University of Science and Technology, Rolla, Missouri, 65409, USA. Corresponding author. E-mail: [Catherine.Johnson@mst.edu](mailto:Catherine.Johnson@mst.edu) Contributing author: [codythomas@mst.edu](mailto:codythomas@mst.edu) Contributing author: [doganf@mst.edu](mailto:doganf@mst.edu)

**Supplementary Data:**


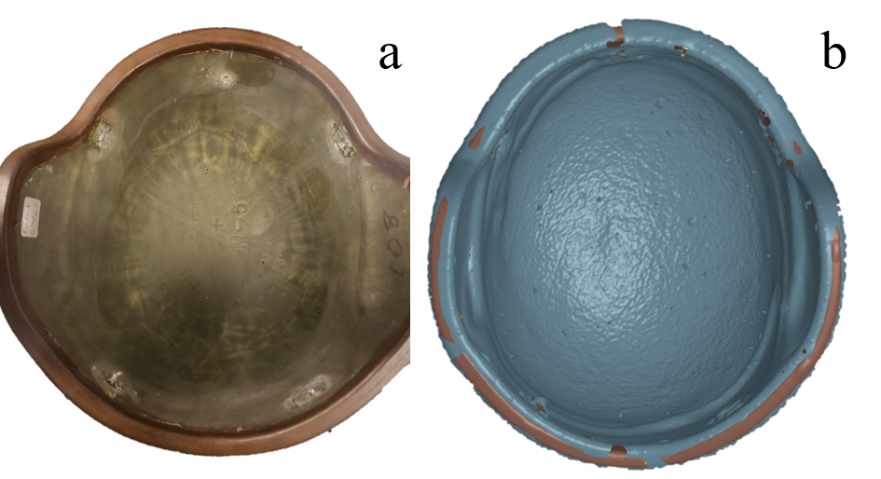


Figure 1. 3D scans comparing (a) the helmet (brown) and (b) the helmet liner (blue).


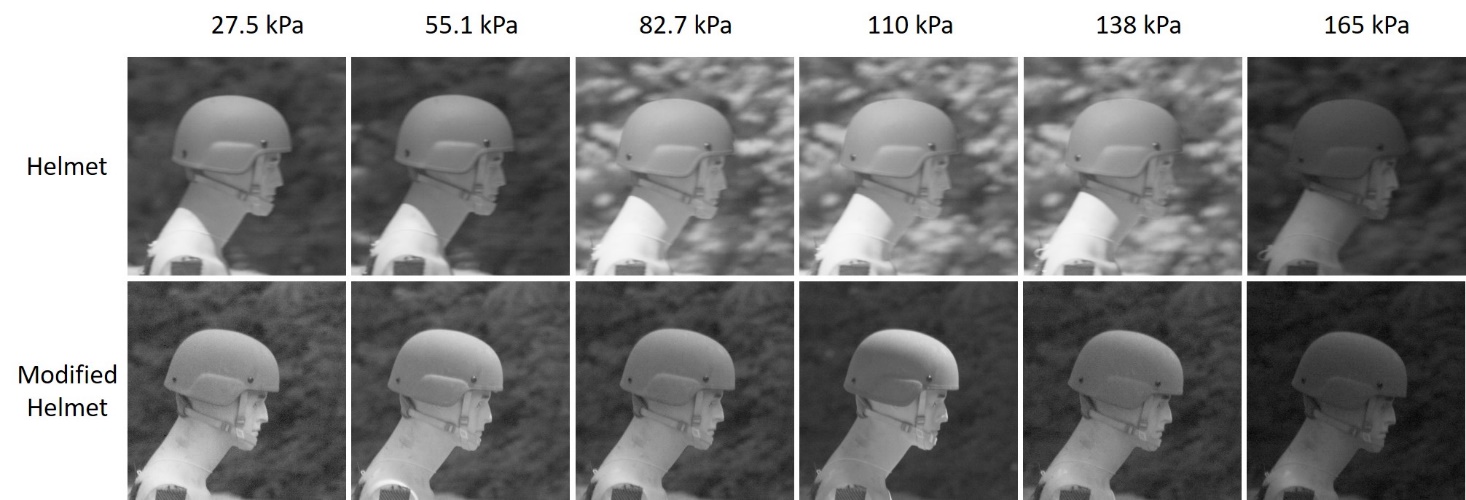


Figure 2. Helmet and helmet liner being worn.

Table 1. Data to create the heat map in Figure 4 which was a 27.5 kPa blast exposure

|  | No Helmet | | Helmet | | Helmet Liner | |
| --- | --- | --- | --- | --- | --- | --- |
| Sensor Number | Peak Pressure (kPa) | Impulse (kPa * ms) | Peak Pressure (kPa) | Impulse (kPa* ms) | Peak Pressure (kPa) | Impulse (kPa * ms) |
| 1 | 82.7 | 21.4 | 82.1 | 23.4 | 82.0 | 21.8 |
| 2 | 59.3 | 21.4 | 60.7 | 24.8 | 30.6 | 22.0 |
| 3 | 39.9 | 18.6 | 24.1 | 22.8 | 21.6 | 22.0 |
| 4 | 35.2 | 21.4 | 32.4 | 32.4 | 25.0 | 29.4 |
| 5 | 26.2 | 15.9 | 31.0 | 26.9 | 21.2 | 21.8 |
| 6 | 21.4 | 20.7 | 51.0 | 35.9 | 25.0 | 30.1 |
| 7 | 26.9 | 19.9 | 55.9 | 27.6 | 26.0 | 19.5 |
| 8 | 24.1 | 18.6 | 48.9 | 16.5 | 53.9 | 16.3 |
| 9 | 36.5 | 15.9 | 37.9 | 19.9 | 35.4 | 23.1 |

Table 2. Data to create the heat map in Figure 5 which was a 165 kPa blast exposure

|  | No Helmet | | Helmet | | Helmet Liner | |
| --- | --- | --- | --- | --- | --- | --- |
| Sensor Number | Peak Pressure (kPa) | Impulse (kPa * ms) | Peak Pressure (kPa) | Impulse (kPa* ms) | Peak Pressure (kPa) | Impulse (kPa * ms) |
| 1 | 510.8 | 141.6 | 510.8 | 109.9 | 511.0 | 98.2 |
| 2 | 344.0 | 118.5 | 434.2 | 106.5 | 227.3 | 93.6 |
| 3 | 183.1 | 72.9 | 135.2 | 92.0 | 103.7 | 87.8 |
| 4 | 155.8 | 62.3 | 146.5 | 115.8 | 130.7 | 101.1 |
| 5 | 118.6 | 52.9 | 147.2 | 116.5 | 113.3 | 94.2 |
| 6 | 98.7 | 66.9 | 241.1 | 122.2 | 110.1 | 98.9 |
| 7 | 146.7 | 72.3 | 269.7 | 72.2 | 205.6 | 78.5 |
| 8 | 113.1 | 70.6 | 246.4 | 71.5 | 208.8 | 56.1 |
| 9 | 171.4 | 54.9 | 155.5 | 102.7 | 178.7 | 70.1 |

Table 3. Peak pressure and impulse for the 55.1 kPa blast exposure

|  | No Helmet | | Helmet | | Helmet Liner | |
| --- | --- | --- | --- | --- | --- | --- |
| Sensor Number | Peak Pressure (kPa) | Impulse (kPa * ms) | Peak Pressure (kPa) | Impulse (kPa* ms) | Peak Pressure (kPa) | Impulse (kPa * ms) |
| 1 | 148.3 | 39.1 | 148.3 | 58.3 | 148.3 | 43.0 |
| 2 | 108.6 | 37.1 | 108.8 | 56.6 | 64.0 | 46.9 |
| 3 | 70.4 | 29.7 | 62.2 | 54.2 | 36.2 | 39.5 |
| 4 | 60.5 | 34.6 | 73.0 | 69.5 | 42.4 | 53.2 |
| 5 | 45.7 | 27.3 | 68.5 | 65.0 | 39.8 | 40.8 |
| 6 | 34.4 | 35.6 | 97.7 | 74.8 | 61.4 | 50.2 |
| 7 | 48.4 | 35.1 | 113.5 | 64.5 | 46.0 | 55.2 |
| 8 | 40.8 | 35.7 | 105.0 | 45.3 | 68.9 | 31.3 |
| 9 | 62.2 | 28.1 | 74.5 | 48.9 | 49.2 | 29.0 |

Table 4. Peak pressure and impulse for the 82.7 kPa blast exposure

|  | No Helmet | | Helmet | | Helmet Liner | |
| --- | --- | --- | --- | --- | --- | --- |
| Sensor Number | Peak Pressure (kPa) | Impulse (kPa * ms) | Peak Pressure (kPa) | Impulse (kPa* ms) | Peak Pressure (kPa) | Impulse (kPa * ms) |
| 1 | 222.2 | 66.1 | 222.2 | 55.9 | 222.2 | 99.2 |
| 2 | 146.3 | 59.3 | 174.9 | 56.2 | 105.2 | 62.1 |
| 3 | 94.5 | 48.0 | 63.0 | 54.3 | 49.2 | 47.3 |
| 4 | 82.0 | 51.6 | 66.5 | 65.8 | 51.8 | 59.4 |
| 5 | 61.2 | 43.7 | 69.7 | 62.7 | 49.2 | 48.5 |
| 6 | 61.4 | 50.6 | 104.8 | 68.0 | 84.8 | 54.7 |
| 7 | 86.9 | 56.9 | 123.9 | 64.1 | 70.6 | 61.8 |
| 8 | 70.9 | 54.2 | 128.1 | 44.2 | 88.9 | 51.8 |
| 9 | 98.2 | 44.7 | 105.5 | 44.6 | 88.4 | 33.8 |

Table 5. Peak pressure and impulse for the 110 kPa blast exposure

|  | No Helmet | | Helmet | | Helmet Liner | |
| --- | --- | --- | --- | --- | --- | --- |
| Sensor Number | Peak Pressure (kPa) | Impulse (kPa * ms) | Peak Pressure (kPa) | Impulse (kPa* ms) | Peak Pressure (kPa) | Impulse (kPa * ms) |
| 1 | 304.3 | 83.7 | 304.3 | 67.8 | 304.3 | 68.7 |
| 2 | 190.0 | 74.7 | 225.4 | 66.8 | 278.2 | 66.1 |
| 3 | 118.2 | 55.7 | 77.7 | 60.9 | 72.0 | 62.1 |
| 4 | 103.8 | 63.9 | 86.8 | 76.5 | 73.8 | 71.8 |
| 5 | 87.1 | 52.8 | 88.3 | 74.6 | 71.7 | 65.4 |
| 6 | 85.7 | 62.2 | 135.5 | 78.2 | 79.4 | 69.5 |
| 7 | 129.1 | 67.4 | 157.6 | 78.2 | 87.9 | 77.7 |
| 8 | 100.6 | 66.0 | 163.9 | 40.8 | 99.2 | 39.1 |
| 9 | 154.4 | 53.4 | 124.9 | 52.2 | 129.1 | 47.8 |

Table 4. Peak pressure and impulse for the 138 kPa blast exposure

|  | No Helmet | | Helmet | | Helmet Liner | |
| --- | --- | --- | --- | --- | --- | --- |
| Sensor Number | Peak Pressure (kPa) | Impulse (kPa * ms) | Peak Pressure (kPa) | Impulse (kPa* ms) | Peak Pressure (kPa) | Impulse (kPa * ms) |
| 1 | 429.9 | 87.4 | 429.9 | 95.8 | 429.9 | 119.2 |
| 2 | 282.7 | 82.7 | 328.4 | 82.5 | 230.5 | 94.8 |
| 3 | 156.6 | 51.7 | 106.3 | 72.0 | 82.0 | 75.2 |
| 4 | 130.5 | 56.6 | 108.2 | 96.6 | 106.9 | 96.5 |
| 5 | 100.8 | 48.1 | 118.8 | 93.5 | 95.7 | 83.9 |
| 6 | 82.3 | 57.9 | 191.1 | 95.8 | 101.9 | 93.0 |
| 7 | 127.8 | 63.8 | 218.0 | 95.5 | 151.1 | 93.6 |
| 8 | 95.9 | 62.3 | 211.9 | 51.9 | 175.7 | 52.4 |
| 9 | 147.9 | 50.1 | 145.9 | 62.8 | 183.0 | 65.8 |


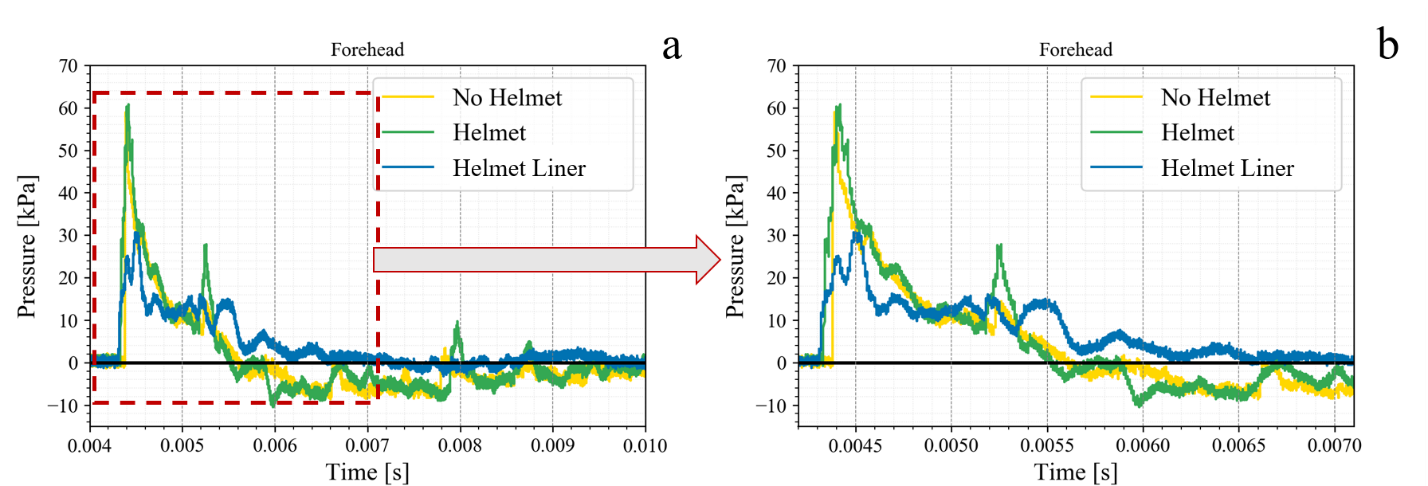


**Figure 3.** Comparison of waveforms for the 27.5 kPa blast exposure without a helmet, with a helmet, and with the viscoelastic material. (a) The entirety of the waveform and section cropped to form (b) the comparison focused on.


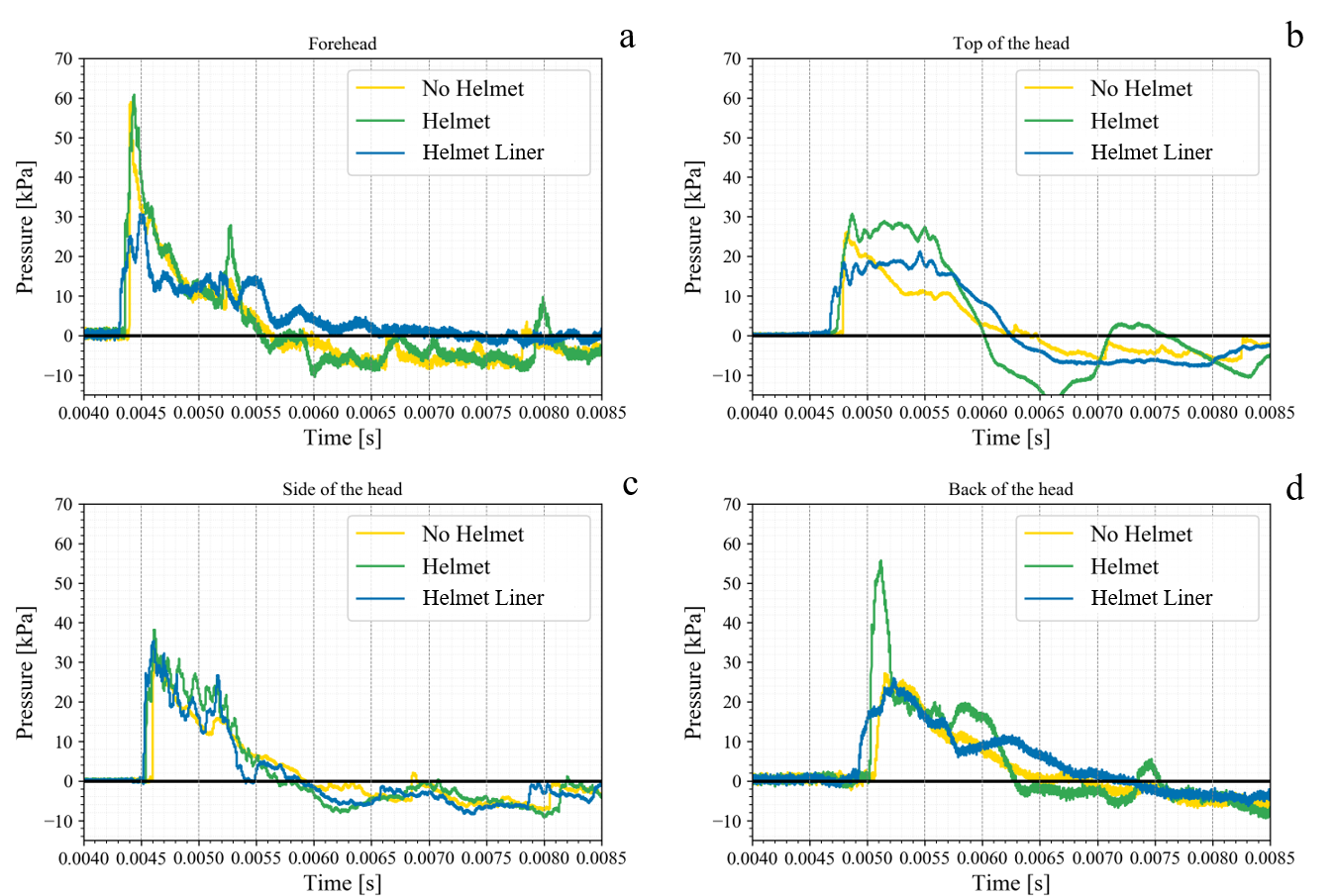


Figure 4. Shock waveforms for the (a) fore head, (b) top of the head, (c) side of the head, and (d) back of the head sensors for the 27.5 kPa blast exposure.


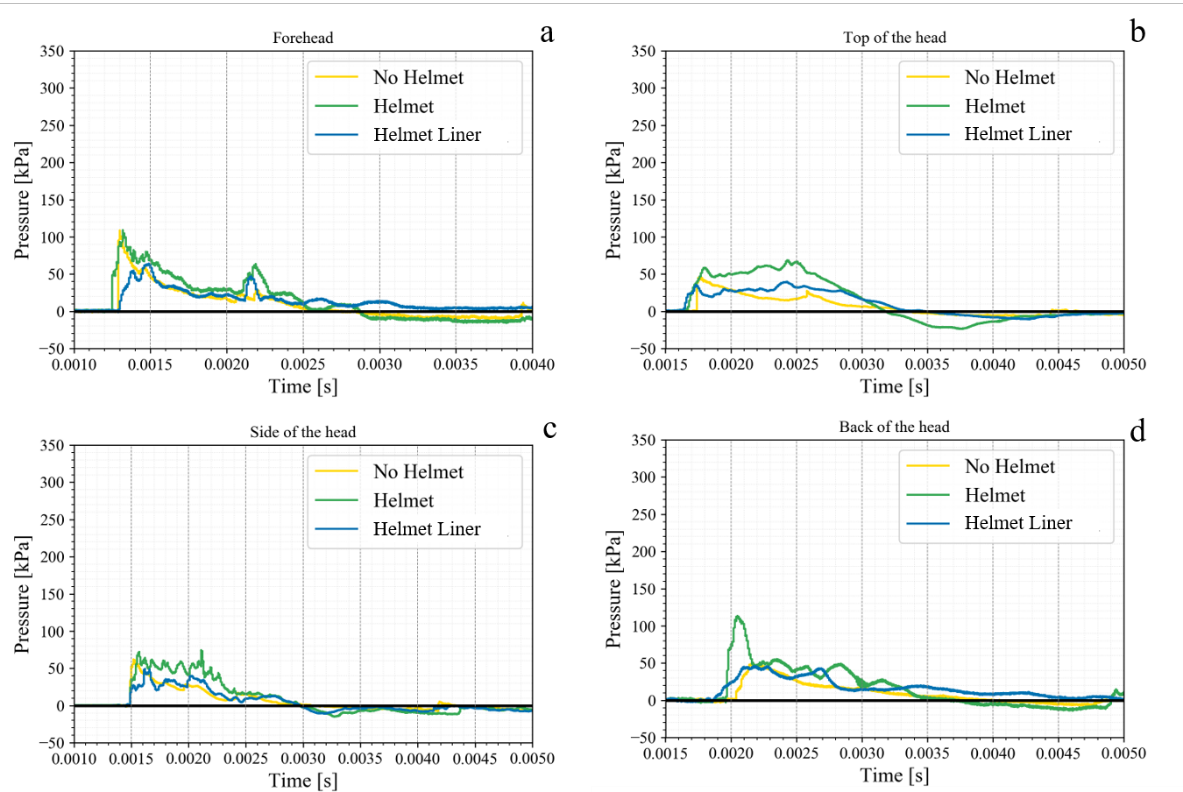


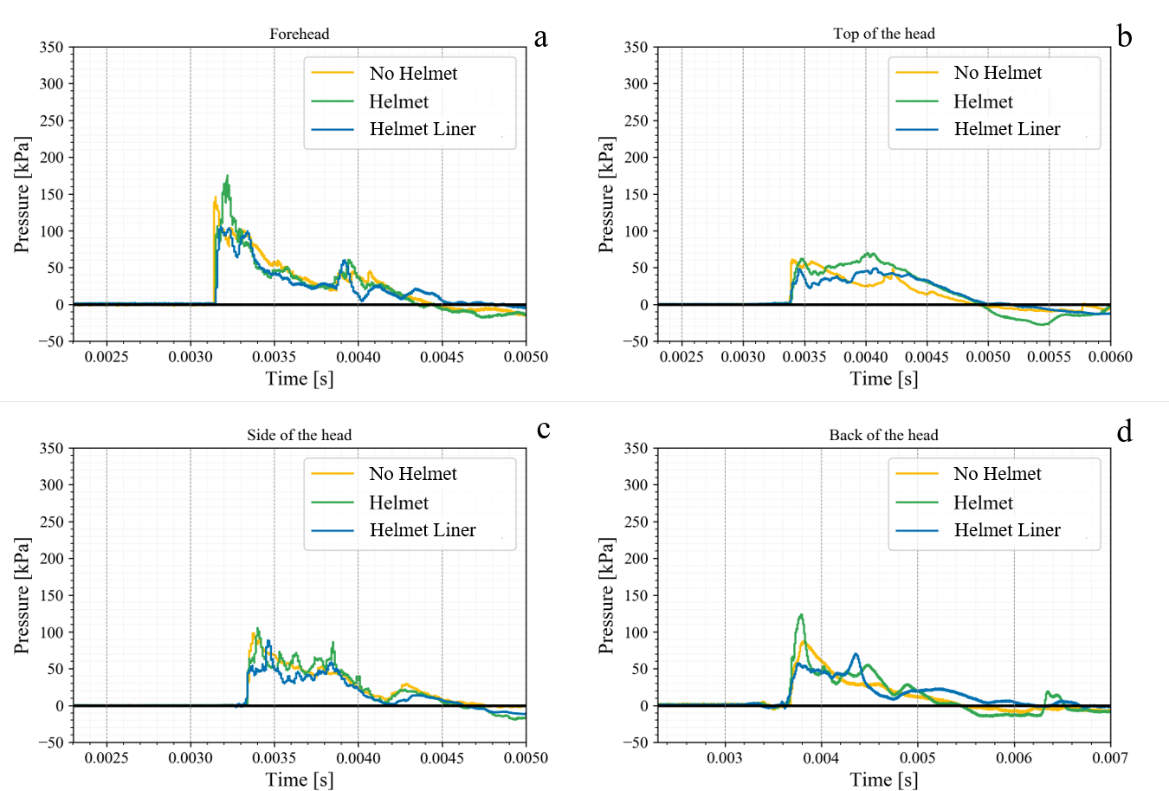
Figure 5. Shock waveforms for the (a) fore head, (b) top of the head, (c) side of the head, and (d) back of the head sensors for the 56.5 kPa blast exposure.

Figure 6. Shock waveforms for the (a) fore head, (b) top of the head, (c) side of the head, and (d) back of the head sensors for the 82.7 kPa blast exposure.


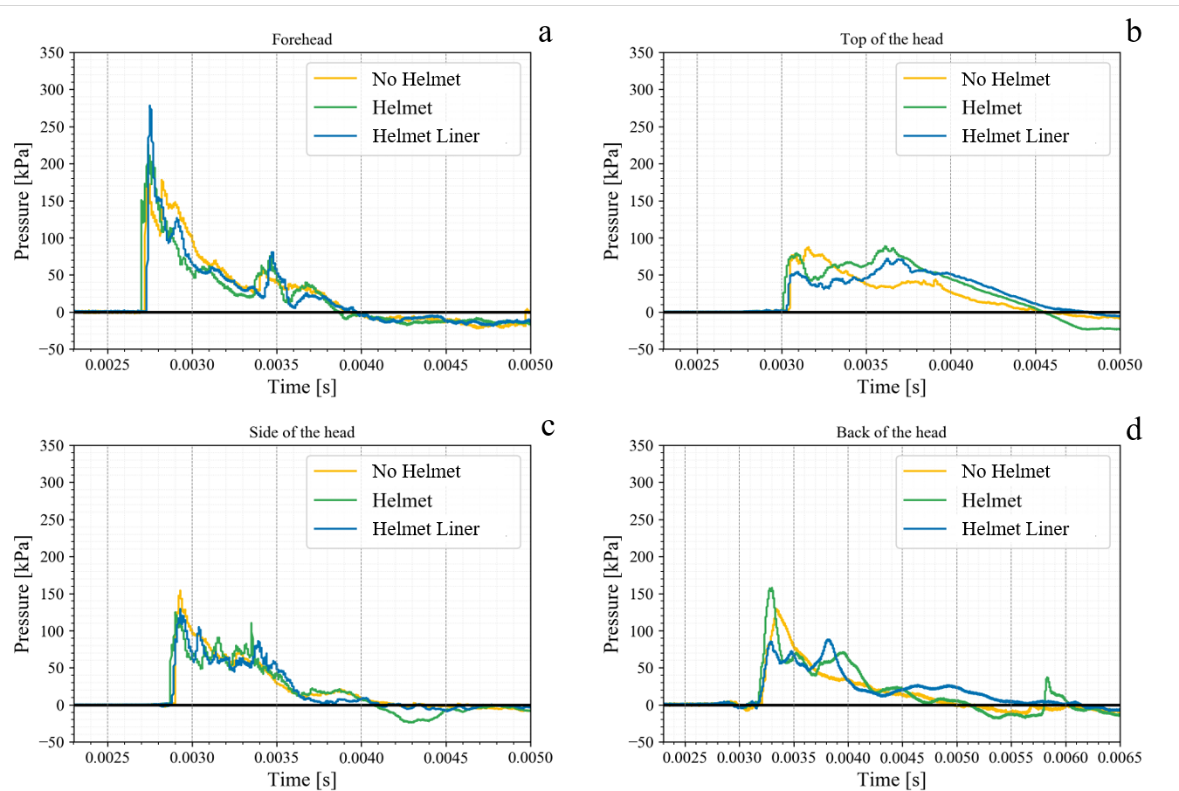


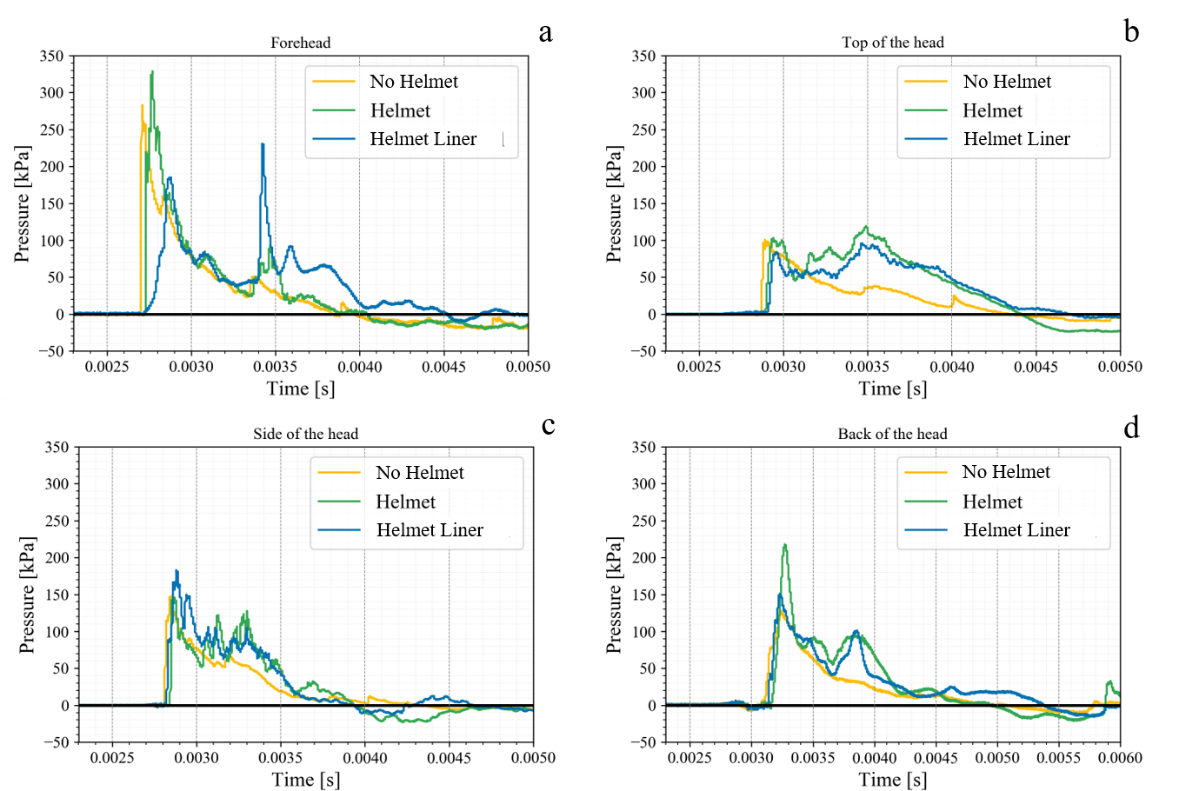
Figure 7. Shock waveforms for the (a) fore head, (b) top of the head, (c) side of the head, and (d) back of the head sensors for the 110 kPa blast exposure.

Figure 8. Shock waveforms for the (a) fore head, (b) top of the head, (c) side of the head, and (d) back of the head sensors for the 137.8 kPa blast exposure.


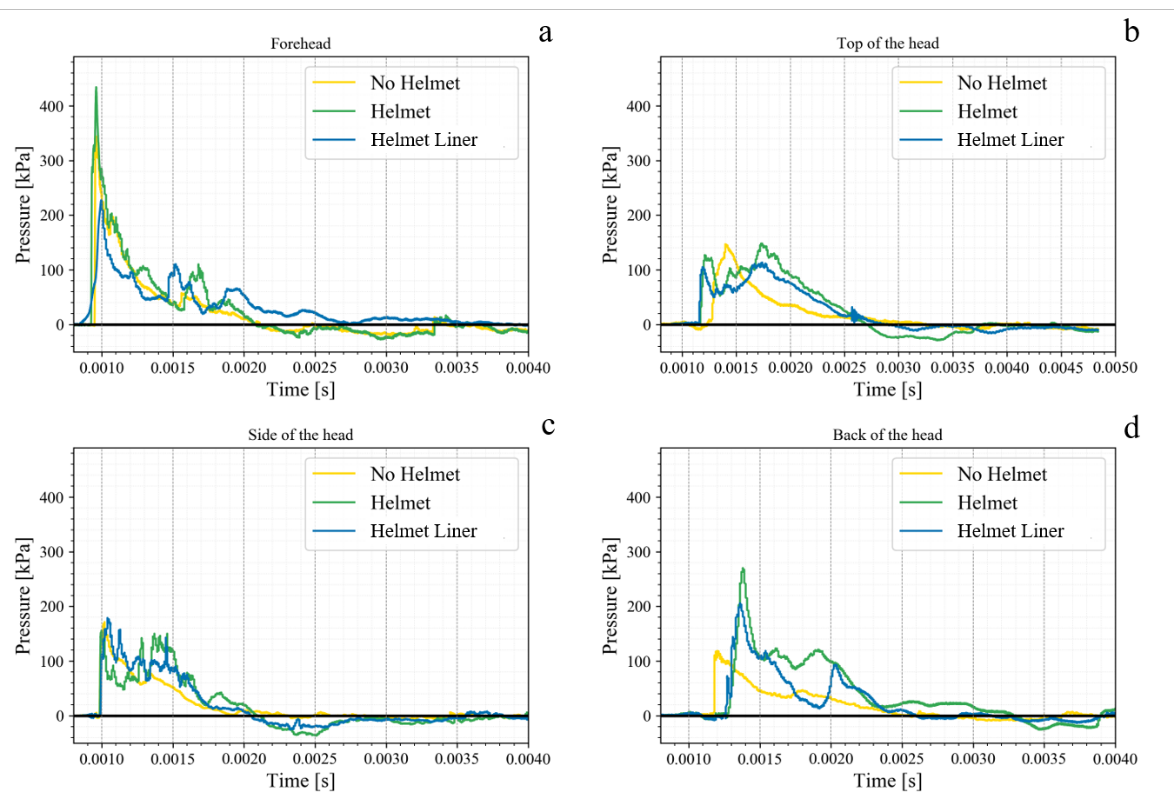


Figure 9. Shock waveforms for the (a) fore head, (b) top of the head, (c) side of the head, and (d) back of the head sensors for the 165 kPa blast exposure.
